# Differences in the Electrophysiological Properties of Mouse Somatosensory Layer 2/3 Neurons *In Vivo* and Slice Stem from Intrinsic Sources Rather than a Network-Generated High Conductance State

**DOI:** 10.1523/ENEURO.0447-17.2018

**Published:** 2018-04-13

**Authors:** Fernando R. Fernandez, Bahar Rahsepar, John A. White

**Affiliations:** Department of Biomedical Engineering, Boston University, Boston, Massachusetts 02215

**Keywords:** dynamic clamp, *in vivo*, input-output, membrane conductance

## Abstract

Synaptic activity *in vivo* can potentially alter the integration properties of neurons. Using recordings in awake mice, we targeted somatosensory layer 2/3 pyramidal neurons and compared neuronal properties with those from slices. Pyramidal cells *in vivo* had lower resistance and gain values, as well as broader spikes and increased spike frequency adaptation compared to the same cells in slices. Increasing conductance in neurons using dynamic clamp to levels observed *in vivo*, however, did not lessen the differences between *in vivo* and slice conditions. Further, local application of tetrodotoxin (TTX) *in vivo* blocked synaptic-mediated membrane voltage fluctuations but had little impact on pyramidal cell membrane input resistance and time constant values. Differences in electrophysiological properties of layer 2/3 neurons in mouse somatosensory cortex, therefore, stem from intrinsic sources separate from synaptic-mediated membrane voltage fluctuations.

## Significance Statement

Neurons *in vivo* are often modeled as being in a high conductance state arising from intense synaptic activity in awake conditions. Outside of the expected reduction in membrane time constant, it remains unclear if other features are altered *in vivo*. Here, we show that cellular properties between mouse somatosensory neurons *in vivo* and slices differ substantially in terms of spike shape, input-output responses, and subthreshold membrane resistance. Increasing the membrane conductance of neurons in slices to match values observed at rest *in vivo*, however, does not generate properties consistent with those observed *in vivo*. Differences in the properties of mouse somatosensory layer 2/3 pyramidal cells *in vivo*, and slices, therefore, are not the product of a network-generated high conductance state.

## Introduction

In cortical neurons synaptic inputs *in vivo* drive variable spike discharge times, smooth spike input-output relationships (Softky and Koch, 1993; Paré et al., 1998; Anderson et al., 2000; Priebe and Ferster, 2008), and reduce the membrane input resistance and time constant of cortical neurons (Bernander et al., 1991; Destexhe and Paré, 1999; Hô and Destexhe, 2000). Extending these results, experiments in slices using artificial changes in membrane conductance, noisy fluctuations, and the presence of neuromodulators have indicated changes to spike voltage threshold, spike frequency adaptation, and the gain of the spike frequency-current relationship (Desai et al., 1999; Cantrell and Catterall, 2001; Cudmore and Turrigiano, 2004; Prescott et al., 2006; Fernandez and White, 2010; Dembrow and Johnston, 2014; Nadim and Bucher, 2014; Wester and McBain, 2014). Membrane conductance and levels of neuromodulation likely differ between *in vivo* and slice conditions. Nevertheless, differences in spike output characteristics between neurons *in vivo* and slices are often modeled as resulting largely from network-generated noisy synaptic inputs that decrease membrane resistance and time constant (Holt et al., 1996; Destexhe et al., 2001, 2003; Chance et al., 2002; Wolfart et al., 2005; Fernandez and White, 2008), rather than differences resulting from neuromodulation or other factors. Only a few studies (Tsuno et al., 2015; Ferrante et al., 2017) have compared spiking properties in identified cell types under both *in vivo* and slice conditions. It remains unknown if the intrinsic spiking properties of neurons that have been established in slice experiments are preserved *in vivo.*

Using visually-guided intracellular whole-cell recordings of somatosensory layer 2/3 pyramidal cells in awake, head-fixed mice, we compared *in vivo* input-output responses and subthreshold membrane properties between slice and *in vivo* layer 2/3 mouse somatosensory pyramidal cells. Compared to pyramidal cells in slices, cells *in vivo* were characterized by reduced input resistance, broader spikes, greater spike frequency adaptation, and a larger voltage dependence in subthreshold membrane input resistance.

To test whether differences between slice and *in vivo* conditions arose from an increase in membrane conductance, we used dynamic clamp in slice recordings to match values observed *in vivo*. Although increasing membrane conductance led to some changes that induced a better match to *in vivo* data, properties associated with spike shape, spike frequency adaptation and the subthreshold current−voltage relationship were altered in a manner inconsistent with *in vivo* measures. Further, the use of tetrodotoxin (TTX) to block synaptic activity *in vivo* minimally altered average membrane input resistance and time constant values of layer 2/3 mouse somatosensory neurons. Taken together, these data indicate fundamental differences between slice and *in vivo* pyramidal cells that are not the result of a high conductance associated with background synaptic activity.

## Materials and Methods

### Ethics statement

All experimental protocols were approved by the Boston University Institutional Animal Care and Use Committee.

### Surgeries

Before surgery, mice (two to six months of either sex) were anaesthetized using ketamine (80–100 mg/kg) and xylazine (8–10 mg/kg). The topical anesthetic bupivicaine (1%) was injected subcutaneously near the site of the surgical incision. The animal also received an injection of the analgesia buprenorphine (0.2–0.5 mg/kg) before surgery. Throughout the surgery, the animal’s body temperature was maintained at 37°C using a water-based heat pad and vitals [heart rate: 125–225 beats/min and estimated oxygen saturation (SPo_2_): 75–99% during surgeries] monitored using a pulse oximeter (Kent Scientific). Ointment was applied to the eyes during surgery to prevent drying. Access to cortical neurons was accomplished using a ∼4 mm^2^ cranial window over cortical area S1 that permitted wide-field visualization and insertion of a standard patch-clamp electrode. A cranial window was centered at 2.5 mm rostral from lamda and 4.5–5 mm lateral from the midline. The cranial window was created with a dental drill and formed within the confines of an aluminum head frame that was anchored to the skull using dental cement and cyanoacrylate. To facilitate electrode access and imaging, the meninges were removed using a small curved needle. To minimize movements associated with mechanical perturbations and prevent exposure of brain tissue, the cranial window was filled with 2% (weight/volume) low melting point agarose, which was dissolved in a sodium chloride solution (0.9% weight/volume). The window was then partially covered using a square glass coverslip. After surgery, the mouse was allowed to recover for a period of at least 2 h so that it was fully awake (heart rate > 350 beats/min) and locomoting. During recordings, the mouse’s head was fixed to a custom aluminum frame and the mouse was partially restrained using flexible 3M Health Care Coban Wrap (3M). Similar to previous work (Constantinople and Bruno, 2011), mice were not habituated to head-fixation; only a brief 30 min period was provided before the recording session. For all recordings, the mouse was placed in a chamber that minimized light and sound and provided a quiet environment. During placement in the recording chamber, the animal was kept warmed using a water-based head pad. For some experiments, vitals were monitored (heart rates were always >400 beats/min and SPo_2_ > 95%).

### Visualizing cortical layer 2/3 neurons and the pipette electrode

To guide electrodes during patch clamp recordings, mice that express the red fluorescent protein tdTomato were used to visualize layer 2/3 excitatory pyramidal cells in somatosensory cortex. C57BL/6J background, CaMKIIa-Cre mice (The Jackson Laboratory, stock #005359; Tsien et al., 1996) were crossed with the lox-stop-lox tdTomato reporter mice (The Jackson Laboratory, stock #007914; Zariwala et al., 2011). CaMKIIa promoter in transgenic mice has been established to drive specific expression of fluorescent protein in layer 2/3 pyramidal cells in S1, with expression in ∼32% of pyramidal cells in layer 2/3 (Wang et al., 2013).

The electrode pipette was visualized using the cyan-green fluorescent dye Alexa Fluor 488 hydrazide (Thermo Fisher Scientific), which was added to the intracellular electrode solution (0.3% weight/volume). Imaging was performed using a two-photon imaging system (Thorlabs) with a mode-locked Ti:Sapphire laser (Chameleon Ultra II; Coherent) set to wavelengths between 920 nm and 950 nm, which was used to excite both the Alexa Fluor 488 and tdTomato using a 20×, NA 1.0 (Olympus) objective lens. Laser scanning was performed using resonant scanners and fluorescence was detected using two photomultiplier tubes (Hamamatsu) equipped with red and green filters to separate emission from Alexa Fluor 488 and tdTomato.

### Viral injection for GCaMP6f expression in CaMKIIa-Cre mice

Eight- to twelve-week-old transgenic CaMKIIa-Cre mice were anesthetized with isofluorane. They were injected with AAV1.Syn.Flex.GCaMP6f.WPRE.SV40 obtained from the University of Pennsylvania Vector Core to express GCaMP6f in the pyramidal cells of layer 2/3 in S1. A total of 200 nl of the virus was injected using a stereotax at 1.1 posterior, 3.3 medial, 300 µm below the Bregma, using a 10-nl syringe (World Precision Instruments) fitted with a 33-gauge needle (NF33BL; World Precision Instruments), at a speed of 40 µl/min controlled via a microsyringe pump (UltraMicroPump 3–4; World Precision Instruments). Postop animals received an intraperitoneal injection of the analgesia Buprenorphin (0.2–0.5 mg/kg) which was continued for 48 h postsurgery every 8–12 h.

### Optical recording of neuronal Ca^2+^ activity

Imaging was performed using a two-photon imaging system (Thorlabs) with a mode-locked Ti:Sapphire laser (Chameleon Ultra II; Coherent) controlled by Thorlabs software. The field of view (500 × 500 µm) was imaged at 29.85 Hz.

### Imaging analysis

Imaging analyses were performed in the Python programming language using a pre-published package (https://github.com/simonsfoundation/CaImAn).

First brain motion was corrected using CaImAn package implementation of rigid motion correction. We identified regions of interests by calcium image source separation based on non-negative matrix factorization that was implemented in CaImAn. This algorithm takes advantage of both spatial and temporal patterns and is able to differentiate overlapping neurons. In the imaged mouse, algorithm identified 88 neurons. ΔF/F was calculated by dividing fluorescent activity of each cell by running average value of its mean background fluorescence. Spiking deconvolution was implemented in this package by fitting an impulse of a second order autoregressive model to the data to estimate the model. This model was then used to solve a non-negative, sparse constrained deconvolution to estimate spiking rate.

### Electrophysiology *in vivo*

The space between the glass coverslip surface and the objective lens was filled with artificial cerebrospinal fluid consisting of the following: 125 mM NaCl, 25 mM NaHCO_3_, 25 mM D-glucose, 2 mM KCl, 2 mM CaCl_2_, 1.25 mM NaH_2_PO_4_, and 1 mM MgCl_2_. A ground electrode, consisting of a silver-chloride wire was placed inside the cranial window. Recordings of intracellular membrane voltage or current were conducted using standard patch clamp electrodes with tips (0.6–1 μm) that had resistance values between 7 and 12 MΩ. Electrodes were pulled using a horizontal puller (Sutter Instruments) using filament, thin-wall glass (Sutter Instruments). Intracellular pipette solution consisted of the following: 120 mM K-gluconate, 20 mM KCl, 10 mM HEPES, 7 mM diTrisPhCr, 4 mM Na_2_ATP, 2 mM MgCl_2_, 0.3 mM Tris-GTP, and 0.2 mM EGTA; buffered to pH 7.3 with KOH.

Patch electrodes were lowered into the cortex using a micromanipulator (Sutter Instruments). Electrodes were lowered into layer 2/3 of cortex ∼150–275 µm below the pial surface. Variable amounts of positive pressure were used to penetrate the agar and pial surface. Under visual guidance, the pressure was then gradually reduced when approaching neurons to minimize mechanical perturbation of cells and increase the likelihood of a successful seal forming. The process of seal formation was constantly monitored using visual cues, as well as 10-mV voltage step to measure seal resistance and the capacitance associated with the glass electrode. On seal formation (>2 GΩ), capacitance compensation was used to eliminate the pipette capacitance during voltage clamp in the on-cell configuration. Small amounts of transient negative pressure were used to break the seal and establish whole cell recordings. Average series resistance values for layer 2/3 pyramidal cells *in vivo* was 48.1 ± 9.2 (*n* = 22). In all current clamp recordings, full bridge balance compensation was used. Throughout recordings, seal resistance was monitored every 2–3 min. Recordings were discarded if changes >10 MΩ were observed. In addition, during current injection, any moment-by-moment change in access resistance was easily detected due to the generation of large-sized voltage artifacts that were only present when injecting current. In all such cases, recordings were discarded. A measured junction potential of 10 mV was subtracted from all voltage traces. Trace signals were amplified and low-pass filtered at 5–10 kHz before being digitized at 10–20 kHz. For LFP recordings, a 0.3–0.6 MΩ electrode filled with ACSF was placed in layer 2/3. LFP recordings were recorded at 20 kHz and filtered between 0.1 and 1000 Hz. All electrophysiology was conducted using a Multiclamp 700B (Molecular Devices) and a Digidata 1550 (Molecular Devices).

For TTX application during *in vivo* recordings, we used a secondary pipette in close proximity to the recording electrode (100–250 μm away) filled with ACSF and containing 50 μM TTX, along with Alexa Fluor 488 hydrazide (0.3% weight/volume) to visualize the delivery of TTX during experiments. To ensure the patch electrode stability, we slowly applied pressure through a syringe until the Alexa Fluor 488 was present in the area around the recording electrode and the cessation of membrane voltage fluctuations.

### Slice preparation

Coronal sections of S1 were prepared from two- to six-month-old mice (same breed of mice as for *in vivo* recordings) of either sex. After anesthetization with isoflurane and decapitation, brains were removed and immersed in 0°C sucrose-substituted artificial cerebrospinal fluid: 185 mM, 2.5 mM KCl, 1.25 mM NaH_2_PO_4_, 10 mM MgCl_2_, 25 mM NaHCO_3_, 12.5 mM glucose, and 0.5 mM CaCl_2_. Coronal slices were cut to a thickness of 400 µm (Leica VT 1200, Leica Microsystems). After the cutting procedure, slices were incubated in ACSF identical to that used in for *in vivo* recordings at 30°C for 20 min before being cooled to room temperature (20°C). After the incubation period, slices were moved to the stage of an infrared differential interference contrast-equipped microscope (Axioscope 2+, Zeiss). All recordings were conducted between 35°C and 36°C. Since the temperature control system could sometimes go above the target temperature, we did not use 37°C.

### Neuron visualization in slices

Neurons in slices were visualized using differential interference contrast microscopy (DIC) and an infrared camera. Visualization of tdTomato-positive CaMKIIa neurons was done through fluorescence microscopy through the use of an LED-based white light source (X-Cite, 120 LED) and excitation and emission filters with center wavelengths of 545 and 605 nm, respectively. We limited our recordings in slices to a region between 150 and 250 μm below the cortical surface to ensure we matched the cortical depth of *in vivo* recordings.

### Electrophysiology in slices

For slice recordings, electrode resistances were between 4 and 6 MΩ, with access resistance values of 27.3 ± 8.8 MΩ. Seal resistance values were always >2 GΩ. Full bridge balance compensation was used for all recordings. Recordings were performed with a current-clamp amplifier (Multiclamp 700B) and data were acquired using a sampling rate of 20 kHz and custom software developed in MATLAB (v. 2015, Mathworks) using the data acquisition toolbox. Other compensation and correction factors were identical to those used *in vivo* recordings. For *in vivo* and slice conditions, pyramidal cell resting values were comparable (*in vivo*: −70 ± 6.7 mV vs slices: −75 ± 5.3 mV), and hence an unlikely source of any potential differences in electrophysiological properties.

### Dynamic clamp

For dynamic clamp experiments, the current-clamp amplifier was driven by an analog signal from an x86 personal computer running Real-Time Experimental Interface (Bettencourt et al., 2008). An increase in linear conductance was introduced via dynamic clamp using the equation:

$$I_{leak}=g_{leak}(V_{mem}-E_{leak})$$

with *V_mem_* representing pyramidal cell membrane voltage and *E_leak_* representing the reversal voltage for the added conductance. For all experiments, *E_leak_* was set to −65 mV. The magnitude of the leak conductance (*g_leak_*) was varied between 4 and 10 nS to achieve a 50% reduction in the membrane input resistance of individual pyramidal cells at resting membrane potentials.

### Data analyses

For all conditions, spike threshold was defined using the peak of the second derivative of the spike wave form. Spike half-width was taken as the width of the spike at voltages corresponding to the half amplitude (mid-point between spike peak amplitude and threshold). Spike rate of rise was defined as the peak of the first derivative associated with the first spike upstroke. Gain was measured using linear regression over the linear range of *f-I* (mean *r* ^2^ = 0.89 ± 0.13) and *f-V* curves (mean *r* ^2^ = 0.93 ± 0.14). Unless otherwise noted, spike parameter measures were taken from the first spike generated. Spike frequency was calculated using the reciprocal of the mean of the interspike intervals generated during 0.5-s-long step depolarizations. Average spiking voltage was calculated as the mean voltage when the cell generated spikes. Dynamic range associated with *f-I* curves was taken as the range in spike discharge frequency between the lowest spike frequency and the spike frequency associated with saturation in the *f-I* curve. Absolute membrane resistance values were calculated from the average slope extracted from *I-V* curves using linear regression. Time constant values in these data were determined using an exponential fit of membrane voltage change (5–10 mV) resulting from a hyperpolarized current step near rest.

To quantify spike frequency adaptation, we took the ratio of the average spike frequency (measured from two interspike intervals) at the end and start of the current pulse, with lower ratio values indicating greater spike frequency adaptation. In some cases, adaption ratio was measured for each current pulse. We quantified changes in spike shape and spike frequency adaptation by taking spike shape and adaptation ratio using the first and last spike frequency value associated with *f-I* curves. For slice and *in vivo* comparisons in pyramidal cells, we used a 0.5-long pulse.

**Figure 1.**
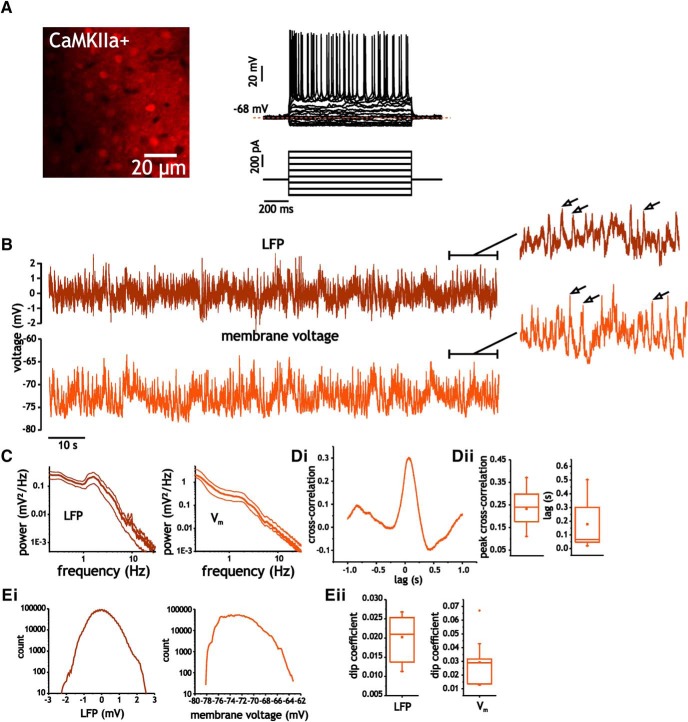
LFP and intracellular membrane voltage traces under awake conditions express unimodal distributions with no evidence of up-down state transitions. ***A***, Representative intracellular recording from a CaMKIIa-positive neuron in layer 2/3 somatosensory cortex of mouse. ***B***, Representative recordings of LFP and intracellular membrane voltage during quiet waking states. The inset shows the last 15 s of the LFP and membrane voltage traces. Arrows indicate the presence of large depolarizations resulting from synchronous synaptic activity. ***C***, Average power spectral density curves (thick lines indicate mean, thin lines indicate standard error from mean). ***D***, Example cross-correlogram (i) of LFP and membrane voltage trace shown in ***B***, as well as average peak cross-correlation coefficients (ii) and absolute lag of peak cross-correlation value (ii, right). ***E***, LFP and membrane voltage traces express unimodal distributions. Example histograms of LFP and membrane voltage traces (i), as well as average dip coefficients for LFP and membrane voltage (ii).

**Figure 2.**
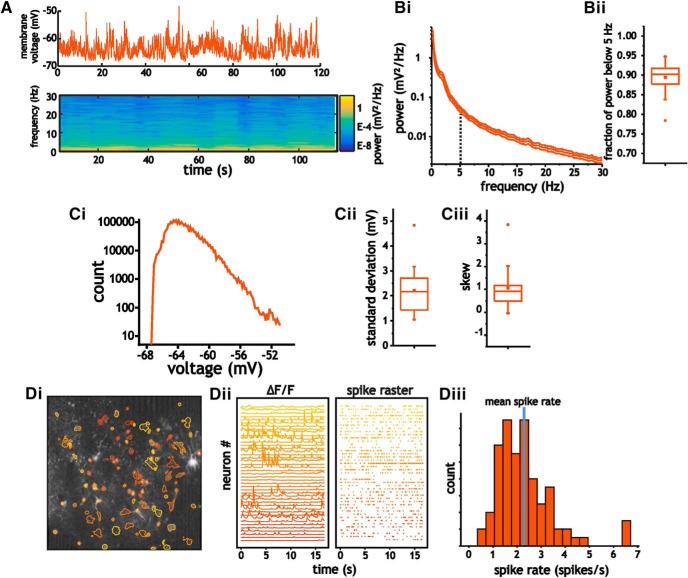
***A***, Example trace (top) and spectrogram (bottom) of membrane voltage fluctuations in pyramidal cells. ***B***, Average power spectra (i) of pyramidal cells and the corresponding fraction of power below 5 Hz (ii). ***C***, Example histogram (i) of intracellular membrane voltage (120-s-long trace) from a pyramidal cell at rest, and the corresponding average standard deviation (ii) and skew coefficient (iii) associated with membrane voltage fluctuations. ***D***, Layer 2/3 pyramidal cells express low spontaneous firing rates under quiet and awake conditions. Example image (i) showing regions of interest used to extract changes in fluorescence (ΔF/F) in CaMKIIa-positive neurons in layer 2/3 somatosensory cortex of mouse (ii, left) and the corresponding spike raster (ii, right) extracted using a deconvolution analysis of changes in fluorescence. Histogram of the distribution of spike rates (iii) from the population of layer 2/3 pyramidal cells that expressed non-zero spike rates.

For power spectral density curves and spectrogram analyses (Figs. 1, 2), we used the Chronux (http://chronux.org/) analysis software toolkit developed in MATLAB (Mathworks). For spectrograms, mean-subtracted, 2-min-long voltages traces were low-pass filtered at 1000 Hz and then analyzed using a multi-taper time-frequency spectrum analysis implemented with the mtspecgramc function included in the Chronux analysis software. The window for the spectrum was set to 10 s. For power spectral density curves, the output from spectrograms was averaged across windows to form a single curve for each data trace. For measures of power, power spectral density curves were integrated between ranges of frequencies specified in the results section. Cross-correlation analyses were conducted using the xcorr function in MATLAB.

### Statistical analyses

Unless otherwise noted, all values are presented as the mean along with the standard deviation. Normality of data points was established using the Shapiro-Wilk test. Positive results from the Shapiro-Wilk test (*p* < 0.05) were used to determine the use of parametric or nonparametric statistical tests noted in the Results section. Data range bars for non-normal distributions use the interquartile range (IQR). Skewness coefficients of voltage and current trace histograms were calculated using the following equation:

$$\gamma =\frac{\frac{1}{n}\sum_{i=1}^{n}(x_{i}-\overset{-}{x}{)}^{3}}{(\frac{1}{n}\sum_{i=1}^{n}(x_{i}-\overset{-}{x}{)}^{2}{)}^{3/2}}.$$

## Results

To compare neuronal responses from specific cell types *in vivo* and slices, we used two-photon imaging to visualize mouse somatosensory layer 2/3 neurons *in vivo* and target for whole-cell intracellular recordings. We used the selective expression of tdTomato under control of the CaMKIIa promoter to visualize and target pyramidal cells (Fig. 1*A*). Average recording depth (cell body location relative to cortical surface) was 182 ± 26 μm. A local field potential (LFP) electrode was placed close to the intracellular recording site (∼250 μm) at the same depth.

Under quiet and awake conditions, LFP recordings were characterized by low frequency power (Fig. 1*C*). As in previous recordings in rat somatosensory cortex during quiet and awake states (Okun et al., 2010), the LFP and intracellular membrane voltage were correlated; peak average cross-correlation coefficient between the LFP and membrane voltage was 0.22 ± 0.08 (*p* < 0.001, Student’s *t* test, *n* = 9; Fig. 1*D*), while the absolute lag at which the peak correlation occurred was 0.18 ± 0.17 s.

Both the LFP and intracellular membrane voltage in pyramidal cells during awake conditions (acquired simultaneously) expressed unimodal distributions, with no signs of up-down state transitions (Fig. 1*Ei*). Correspondingly, the Hartigan’s dip test (Hartigan and Hartigan, 1985) for unimodality applied to LFP and membrane voltage traces expressed values of 0.02 ± 0.01 and 0.03 ± 0.02, respectively, with neither average values reaching significance for a nonunimodal distribution (*p* = 0.64 and *p* = 0.42, Hartigan’s dip test, *n* = 9; Fig. 1*Eii*). This is consistent with previous recordings in mouse and rat somatosensory cortex under awake conditions (Crochet and Petersen, 2006; Okun et al., 2010). Thus, while under anesthetized conditions the LFP and membrane voltage trace in somatosensory cortex often express discrete up-down states that are discernable statistically using histograms (Waters and Helmchen, 2006), we saw no evidence for these transitions in our recordings (Fig. 1*B*,*E*; also see Fig. 2). Nevertheless, visual inspection of membrane voltage traces and the LFP indicated large synchronous events (Fig. 1*B*, insets). These events were sufficiently oscillatory to generate a peak in the power spectra of the LFP near 2 Hz (Fig. 1*C*) and are consistent with quiet and awake states. This indicates that conditions in our recordings were associated with a synchronous and lower conductance state similar to that described by Tan et al. (2014), in which membrane voltage fluctuations are driven by sparse and synchronous volleys of synaptic activity.

As in past studies of layer 2/3 somatosensory cortical neurons in awake mice (Crochet and Petersen, 2006; Crochet et al., 2011; Yamashita et al., 2013), voltage fluctuations during quiet waking states in pyramidal cells (Fig. 2*Ai*) were associated with low frequency power. Approximately ∼ 90% of power was contained below 5 Hz [0.89 ± 0.04 (SEM), *n* = 21; Fig. 2*B*], with average standard deviation of membrane voltage fluctuations of 2.2 ± 0.89 mV (Fig. 2*Cii*). Fluctuations were also characterized by non-Gaussian distributions that contained positive skews. Histograms of membrane voltage traces expressed average skew coefficients of 1.1 ± 0.67 (IQR), which were significantly different from the near-zero value expected from a Gaussian distribution (Wilcoxon rank sum test, *p* < 0.001, *n* = 21; Fig. 2*Ciii*).

Finally, we also quantified the average firing rate of pyramidal cells under quiet and awake conditions. Using whole-cell patch recordings we found that most cells did not fire. However, using a deconvolution analysis of changes in fluorescence (ΔF/F; see Materials and Methods) from GCaMP6f to extract firing rates from spontaneous Ca^2+^ activity indicated rates substantially above zero (Fig. 2*D*). Using this analysis, the average firing rate for pyramidal cells was 2.3 ± 1.2 spikes/s (*n* = 88 cells; Fig. 2*Diii*). This value is within those acquired in layer 2/3 using Ca^2+^ imaging (O’Connor et al., 2010) but higher than those acquired using intracellular recordings (Crochet and Petersen, 2006).

In summary, and consistent with previous recordings in mice and rats under awake conditions, membrane voltage traces in pyramidal cells expressed standard deviations in the range of 2 mV, were dominated by low frequency power and expressed unimodal and skewed distributions.

### Electrophysiological properties of pyramidal cells differ between *in vivo* and slice conditions

The membrane voltage fluctuations observed above are consistent with a barrage of background synaptic activity often associated with *in vivo* conditions. These conditions are often modeled in slices and computational studies as generating a higher conductance state than those in slices (Chance et al., 2002; Destexhe et al., 2003; Fernandez and White, 2008; Kumar et al., 2008). Furthermore, increases in membrane conductance of neurons in slices using dynamic clamp have been shown to reduce gain, increase spike frequency adaptation, and increase spike voltage threshold (Prescott et al., 2006; Fernandez and White, 2010). These conditions could alter pyramidal cell properties under awake conditions. To address this, we compared results from *in vivo* recordings with those from the same cell type in coronal slices of mouse S1.

**Figure 3.**
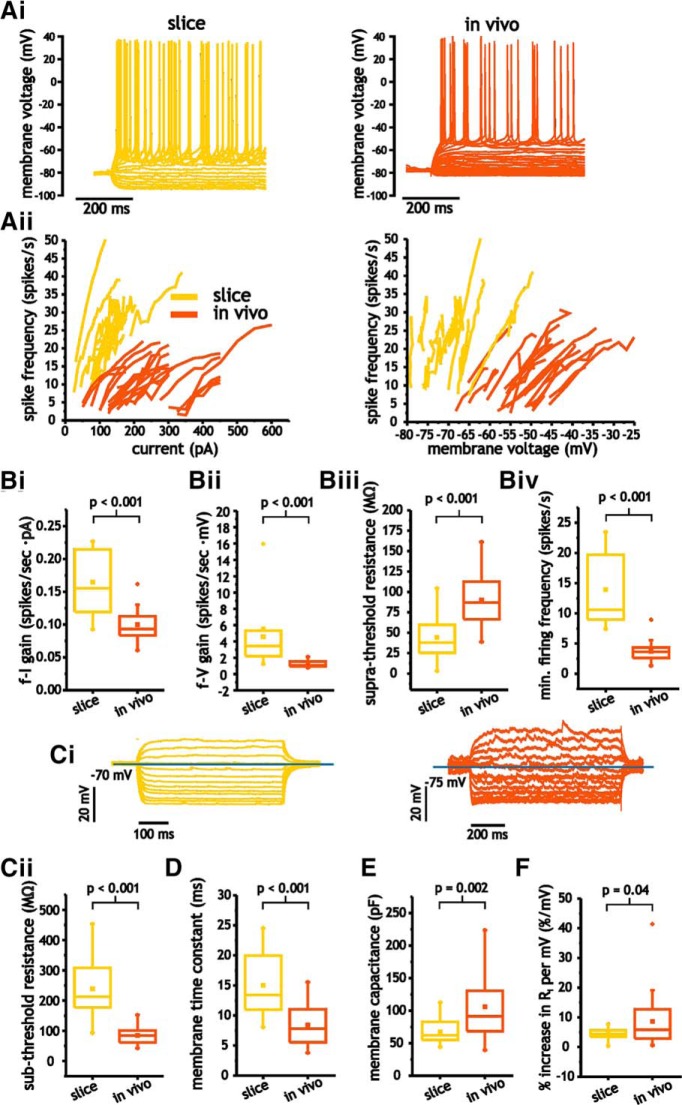
Pyramidal cells express significant differences in electrophysiological properties between *in vivo* and slice conditions. ***A***, Representative voltage traces from pyramidal cells in response to step depolarizations (i) and *f-I* and *f-V* curves (ii) for both *in vivo* and slice neurons. ***B***, Comparison of average *f-I* (i) and *f-V* (ii) gain, as well as suprathreshold membrane resistance (iii) and minimum spike firing frequency (iv) in pyramidal cells *in vivo* and in slices. ***Ci***, Representative subthreshold voltage traces from pyramidal cells in response to step depolarizations *in vivo* and slice neurons. ***Cii–E***, Comparison of average membrane input resistance (***Cii***), time constant (***D***), and membrane capacitance (***E***) in pyramidal cells under *in vivo* and slice conditions. ***F***, Comparison of average percentage increase in subthreshold membrane resistance *in vivo* and slice conditions.

For pyramidal cells, comparisons of *f-I* and *f-V* curves indicated that neurons in slices expressed significantly larger gain values than those *in vivo* [*f-I*: 0.16 ± 0.05 vs 0.10 ± 0.02 spikes/s·pA, *p* < 0.001, Student’s *t* test, *n* = 11 and *n* = 22; *f-V*: 4.6 ± 3.1 vs 1.2 ± 0.60 (IQR) spikes/s·mV, *p* < 0.001, Mann–Whitney *U* test, *n* = 11 and *n* = 22; Fig. 3*A*, *Bi*,*ii*). We also compared the average resistance during spiking (suprathreshold resistance). This measure includes spikes and hence relates how average spike shape and mean voltage between spikes changes as a function of input current. Suprathreshold resistance was significantly lower in slices than *in vivo*, with average values of 44.4 ± 31.9 and 90.1 ± 30.5 MΩ, respectively (*p* < 0.001, Student’s *t* test, *n* = 11 and *n* = 22; Fig. 3*Biii*). Finally, the minimum onset spike frequency associated with *f-I* and *f-V* curves was significantly higher in slices than *in vivo* (13.9 ± 6.8 vs 3.7 ± 6.2 spikes/s, *p* < 0.001, Student’s *t* test, *n* = 11 and *n* = 22; Fig. 3*Biv*).

Slice and *in vivo* cells also differed in terms of subthreshold resistance. In slices, pyramidal cells expressed much higher average subthreshold resistance (238 ± 99 vs 84 ± 29 MΩ, *p* < 0.001, Student’s *t* test; Fig.3*Cii*) and time constants (15.0 ± 5.0 vs 8.4 ± 3.4 ms, *p* < 0.001, *n* = 11 and *n* = 22; Fig. 3*D*). Capacitance measures (calculated from our measures of time constant and resistance) also showed significantly lower values in slices than *in vivo* (67.2 ± 19.6 vs 106 ± 44.0 pF, *p* = 0.002, Student’s *t* test; Fig. 3*E*). Thus, while the *in vivo* conditions were consistent with sparse and synchronous synaptic activity associated with lower conductance states, pyramidal cells *in vivo* still expressed much lower membrane input resistance than those in slices.

Inspection of individual *I-V* relationships also revealed that neurons expressed nonlinear *I-V* curves. Under both conditions, the slope (resistance) increased with depolarization. To quantify this, we took the average resistance across the three most hyperpolarized measures of resistance (starting at −85 mV) and compared the value with the average acquired at the three most depolarized values (ending at −55 mV). The increase in membrane resistance observed was lower in slices than *in vivo* (4.3 ± 2.0%/mV vs 8.6 ± 8.9 ms, *p* = 0.04, Student’s *t* test, *n* = 11 and *n* = 22; Fig. 3*F*). In summary, our measures of spike input-output and subthreshold membrane properties indicated significant differences, with little overlap in these properties between *in vivo* and slice recordings.

### Layer 2/3 pyramidal cells express broader spikes and greater spike frequency adaptation under *in vivo* conditions

To uncover mechanisms that could account for differences between slice and *in vivo* conditions, and whether they could arise from increasing membrane conductance associated with *in vivo* conditions, we examined factors associated with excitability as reflected in the spike shape. This included the rate of rise, voltage threshold, and spike half-width. To control for the effects of spike rate on these measures, we used spikes generated at comparable discharge frequencies (∼10–15 spikes/s for both conditions).

**Figure 4.**
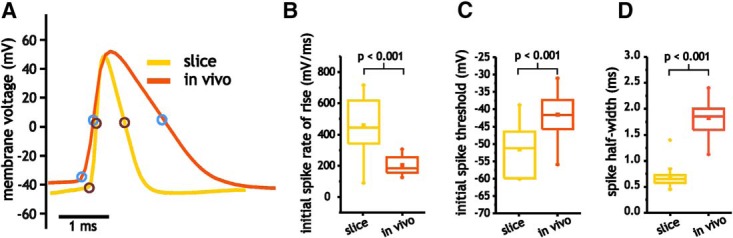
Comparison of pyramidal cell spike shape *in vivo* and in slices. ***A***, Example of spike shape and the corresponding points associated with threshold and half-width *in vivo* and slices. ***B–D***, Plot of average initial spike rate of rise (***B***), spike threshold (***C***), and half-width (***D***) for pyramidal cells *in vivo* and in slices.

All three spike parameters differed substantially between pyramidal cells in slices and those measured *in vivo* (Fig. 4). The maximum spike rate of rise, which is proportional to the Na^+^ current amplitude underlying the upstroke in spike generation (Hodgkin and Katz, 1949; Hondeghem, 1978; Strichartz and Cohen, 1978), was much higher in slices than *in vivo* (459 ± 183 vs 204 ± 57.0 mV/ms, *p* < 0.001, Student’s *t* test, *n* = 11 and *n* = 22; Fig. 4*B*). Consistent with the difference in the rate of rise, spike voltage threshold was also lower in neurons from slices than those recorded *in vivo* (−51.5 ± 7.5 vs −41.6 ± 6.8 mV, *p* < 0.001, Student’s *t* test, *n* = 11 and *n* = 22; Fig. 4*C*). In slices, spikes were also substantially narrower than those *in vivo*, with average half-widths of 0.71 ± 0.26 ms compared with 1.82 ± 0.34 ms under *in vivo* conditions (*p* < 0.001, Student’s *t* test, *n* = 11 and *n* = 22; Fig. 4*D*). These measures suggest that differences in gain between slice and *in vivo* pyramidal cells are related to a reduction in Na^+^ current availability during spiking and its impact on voltage threshold. Alternatively, the wider spikes *in vivo* are also consistent with lower levels and/or slower K^+^ currents during spike repolarization. Together, these factors can potentially account for the lower maximum spike discharge frequency and gain observed *in vivo*.

**Figure 5.**
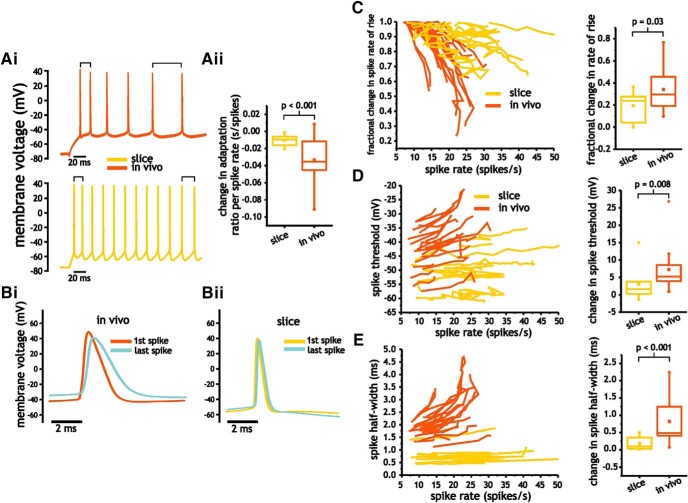
Pyramidal cells *in vivo* express greater changes in spike shape and spike frequency adaptation with increasing spike discharge frequency. ***A***, Differences in spike frequency adaptation between *in vivo* and slice pyramidal cells. Representative voltage trace indicating greater adaptation in *in vivo* pyramidal cells (i) and plot of average change in adaptation ratio per spike rate (ii). ***B***, Spike frequency-dependent changes in pyramidal cell spike shape *in vivo* (i) and in slices (ii). ***C–E***, Plots of fractional change in spike rate of rise (***C***), voltage threshold (***D***), and half-width (***E***) as a function of spike discharge frequency along with the corresponding averages changes (between initial, low spike discharge rates, and highest discharge rates) for each of the spike measures.

An increase in spike frequency adaptation induced by lower Na^+^ current availability could account for a lower maximum spike discharge frequency and gain. To quantify spike frequency adaptation, we took the ratio of the average spike frequency (estimated from two interspike intervals) at the end and start of the current pulse. The adaption ratio was measured for each current pulse with at least five spikes, with lower ratio values indicating greater spike frequency adaptation. The majority of neurons in slice and *in vivo* expressed some degree of spike frequency adaptation. Measures *in vivo*, however, showed greater changes in the spike frequency adaptation ratio with increasing firing rate (−0.0334 ± 0.03 vs −0.0104 ± 0.01 s/spikes, *p* < 0.001, Student’s *t* test; Fig. 5*A*).

If changes in spike shape associated with lower Na^+^ current availability establish an increase in adaptation, spike shape parameters should also be frequency dependent, and express greater changes *in vivo* than in slices. Consistent with this hypothesis, we observed larger changes in spike shape with increasing spike discharge frequency *in vivo* (Fig. 5*B–E*). Between the lowest and highest spike discharge rate, changes in the spike rate of rise (normalized to the first spike) and spike threshold were both greater in *in vivo* than in slices; rate of rise changed by 0.34 ± 0.19 and 0.19 ± 0.13 (Student’s *t* test, *p* = 0.03, *n* = 11 and *n* = 22; Fig. 5*C*), while spike threshold depolarized by 7.2 ± 6.1 and 3.1 ± 4.9 mV, respectively (*p* = 0.008, Mann–Whitney *U* test, *n* = 11 and *n* = 22; Fig. 5*D*). Changes in spike half-width were also greater *in vivo* than in slices, with half-width increasing by 0.82 ± 0.84 (IQR) ms *in vivo* and 0.18 ± 0.32 (IQR) ms in slices (Mann–Whitney *U* test, *p* < 0.001, *n* = 11 and *n* = 22; Fig. 5*E*).

### Increasing membrane conductance in slice pyramidal cells generates modest changes in spike shape and linearizes the subthreshold *I-V* relationship

Using dynamic clamp, we tested the hypothesis that differences between slice and *in vivo* pyramidal cell output could arise from an increase in membrane conductance. In particular, previous studies have indicated that adding a linear conductance to cells in slices can fundamentally alter input-output responses and spike shape (Prescott et al., 2006; Fernandez and White, 2010). Although our dynamic clamp implementation is limited to changing membrane resistance at soma, our measures indicated significant differences between *in vivo* and slice membrane input resistance as measured at the cell body. Thus, a reduction in membrane input resistance in slice pyramidal cells can be used to test if an increase in membrane conductance at the soma reproduces feature observed *in vivo*.

**Figure 6.**
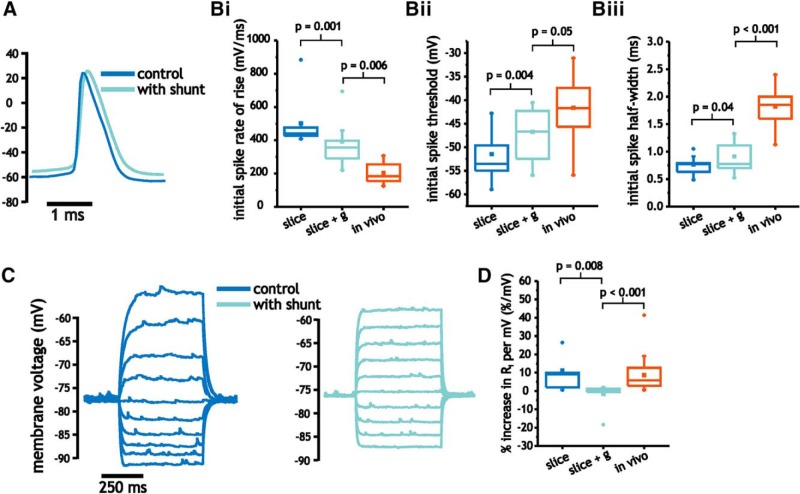
Comparison of pyramidal cell spike shape and *I-V* curve under control and with added conductance. ***A***, Example of spike shape under control and with added conductance. ***B***, Plot of average initial spike rate of rise (i), spike threshold (ii), and half-width (iii) for pyramidal cells under control and with added conductance, as well as under *in vivo* conditions. ***C***, Example traces of subthreshold membrane voltage in response to step depolarization between −90 and −50 mV under control and with added conductance. ***D***, Comparison of average percentage increase in subthreshold membrane resistance under control and with added conductance, as well as under *in vivo* conditions.

We reduced membrane input resistance by ∼50% (142 ± 68.9 to 70.1 ± 17.2 MΩ; 14.8 ± 4.7 vs 8.5 ± 2.7 ms) in slice pyramidal cells through the addition of a linear conductance with a magnitude between 6 and 10 nS, which corresponded to the average differences observed between slices and *in vivo* recordings. We started by measuring the impact of increasing membrane conductance on spike shape. As shown, membrane conductance led to lower spike rates of rise [502 ± 62.1 vs 392 ± 114 (IQR) mV/ms, *p* = 0.001, paired Wilcoxon rank sum test, *n* = 8; Fig. 6*Bi*], more depolarized spike thresholds (−51.5 ± 5.1 vs −46.7 ± 5.7 mV/ms, *p* = 0.004, paired Student’s *t* test, *n* = 8; Fig. 6*Bii*), and larger half-widths (0.77 ± 0.17 vs 0.91 ± 0.27 ms, *p* = 0.04, paired Student’s *t* test, *n* = 8; Fig. 6*Biii*). Although the direction of these changes were consistent with differences observed between slices and *in vivo* measures, the magnitudes of change were too small to match values observed *in vivo* (Student’s *t* test, *p* < 0.01, *n* = 8 and *n* = 22; Fig. 6*Bi*,*iii*). Only the changes in spike threshold came close to that measured *in vivo* (Student’s *t* test, *p* = 0.05, *n* = 8 and *n* = 22; Fig. 6*Bii*).

Finally, we compared the effects of increasing membrane conductance on the non-linear increase in membrane resistance observed between −85 mV and spike threshold. As before, we observed that membrane input resistance increased as the cell was depolarized (Fig. 6*C*). The addition of membrane conductance largely eliminated the increase [11.2 ± 12.5 vs −1.7 ± 1.4 (IQR) %/mV, *p* = 0.008, paired Wilcoxon rank sum test, *n* = 8; Fig. 6*C*,*D*], and consequently widened the difference between *in vivo* and slice pyramidal cell data (Mann–Whitney *U* test, *p* < 0.001, *n* = 8 and *n* = 22; Fig. 6*D*).

### Increasing membrane conductance in slice pyramidal cells does not account for differences in spike input-output curves and adaptation

Stronger spike frequency adaptation, associated with cumulative Na^+^ current inactivation and/or activation of K^+^ currents can result from an increase in membrane conductance associated with *in vivo* conditions that increase spike voltage threshold (Prescott et al., 2006; Fernandez and White, 2010). Given the depolarization of spike voltage noted previously (Fig. 6*B*), adding membrane conductance can potentially increase spike frequency adaptation and lower *f-I* and *f-V* curve gain values.

**Figure 7.**
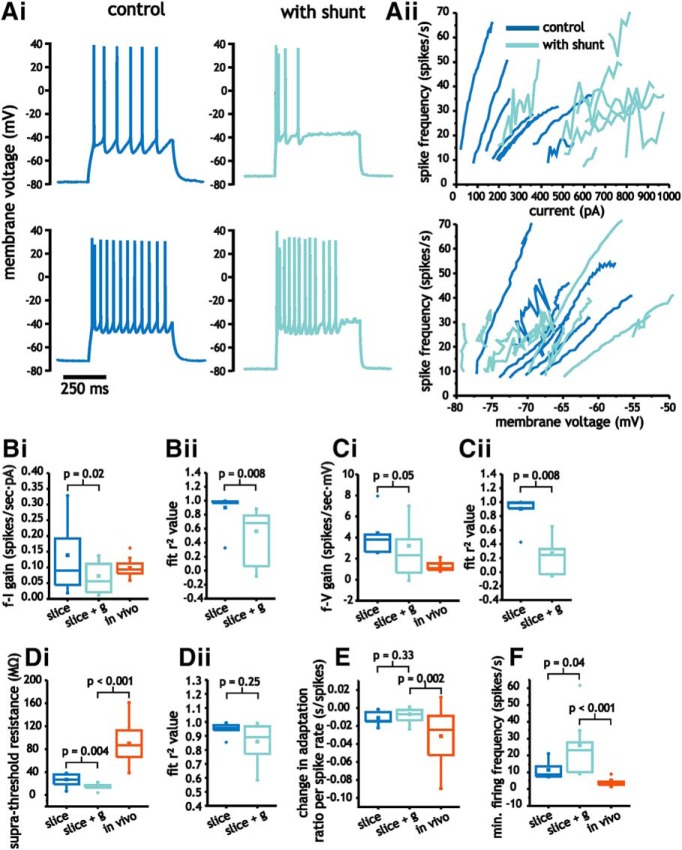
Increasing membrane conductance in pyramidal cells to levels observed *in vivo* modulates input-output responses. ***A***, Representative voltage traces from pyramidal cells (i) in response to step depolarization, along with *f-I* and *f-V* curves (ii) for control conditions and with added conductance. ***B***–***D***, Comparison of average *f-I* (***B***) and *f-V* (***C***) gain, as well as suprathreshold membrane resistance (***D***) between slice under control and with added conductance, as well as those acquired *in vivo*. ***E***, ***F***, Changes in minimum spike firing frequency (***E***) and spike frequency adaptation (***F***) associated with *f-I* and *f-V* curves under slice (control and added conductance) and *in vivo*.

Increasing membrane conductance had complex effects on *f-I* and *f-V* curves. Although average slope values of *f-I* curves decreased with added conductance (0.14 ± 0.10 vs 0.07 ± 0.05 spikes/s·pA, paired Student’s *t* test, *p* = 0.02, *n* = 8; Fig. 7*Bi*), linear fits of *f-I* curves under these conditions were poor. The average *r* ^2^ value associated with linear regression fits decreased from 0.90 ± 0.02 to 0.56 ± 0.50 (IQR; *p* = 0.008, paired Wilcoxon rank sum test, *n* = 8; Fig. 7*Bii*). For the *f-V* curve, average slope values were unchanged [4.4 ± 3.2 vs 3.2 ± 4.2 (IQR) spikes/s·mV, *p* = 0.05, paired Wilcoxon rank sum test, *n* = 8; Fig. 7*Ci*]. As in *f-I* curves, however, adding conductance lowered the average *r* ^2^ values from 0.90 ± 0.07 to 0.27 ± 0.41 (IQR; *p* = 0.008, paired Wilcoxon rank sum test, *n* = 8; Fig. 7*Cii*).

Analysis of the suprathreshold resistance revealed a significant decrease with added conductance (27.0 ± 10.8 vs 14.5 ± 5.4 MΩ, paired Student’s *t* test, *p* = 0.004, *n* = 8; Fig. 7*Di*). As a result, compared to values acquired *in vivo*, suprathreshold resistance was even more different than those under control conditions (Student’s *t* test, *p* < 0.001, *n* = 8 and *n* = 22; Fig. 7*Di*). Unlike the *f-I* and *f-V* curve, the *r* ^2^ values associated with linear fits of suprathreshold *I-V* curves remained high and did not significantly change with added conductance [0.95 ± 0.03 vs 0.86 ± 0.20 (IQR), *p* = 0.25, paired Wilcoxon rank sum test, *n* = 8; Fig. 7*Dii*]. The lower slope value indicates that increasing membrane conductance compresses the voltage range associated with spiking rather than expanding as was the case *in vivo*.

The relationship between spike frequency adaptation and firing rate was not altered by increasing membrane conductance (0.012 ± 0.007 vs 0.007 ± 0.008 s/spikes, paired Student’s *t* test, *p* = 0.33, *n* = 8; Fig. 7*E*). Thus, spike frequency adaptation *in vivo* continued to increase significantly more with firing rate than pyramidal cells in slices with added conductance (Student’s *t* test, *p* = 0.002, *n* = 8 and *n* = 22; Fig. 7*E*).

Finally, conductance induced an increase in the minimum onset frequency associated with *f-I* and *f-V* curves [11.5 ± 7.1 vs 26.1 ± 18.9 (IQR) spikes/s, *p* = 0.04, paired Wilcoxon rank sum test, *n* = 8; Fig. 7*F*), which increased the difference between *in vivo* and slice data (Mann–Whitney *U* test, *p* < 0.001, *n* = 8 and *n* = 22; Fig. 7*F*). Adding conductance, however, did not increase the maximum firing rate associated with input-output curves (41.4 ± 16.7 vs 36.8 ± 15.2 spikes/s, paired Student’s *t* test, *p* = 0.38, *n* = 8). The decrease in *f-I* curve gain, therefore, resulted from an increase in minimum firing rate rather than an increase in spike frequency adaptation.

In summary, for *f-I*, *f-V* and suprathreshold curves, changes induced by increasing membrane conductance either fundamentally changed the relationship between input and output measures, or the direction of change was the opposite of the observed differences between *in vivo* and slice data. Along with a lack of change in spike frequency adaptation, limited changes in spike shape, and a linearization of subthreshold *I-V* curves, these results indicate that an increase in linear membrane conductance, as expected from intense background synaptic activity, is insufficient to account for the differences in integration properties observed between *in vivo* and slice pyramidal cells in layer 2/3 mouse somatosensory pyramidal cells.

### Silencing synaptic activity minimally impacts resting membrane conductance

Our results suggested that an increase in linear conductance brought about through synaptic activity *in vivo* is unlikely to account for differences between slice and *in vivo* conditions. In particular, the greater increase in membrane resistance with depolarization observed *in vivo* suggests a limited contribution from large mean linear synaptic conductances that would be expected from ongoing AMPAergic or GABAergic synaptic activity. Given this, we hypothesized that blocking synaptic activity *in vivo* would not alter membrane conductance and indicate that the low average membrane input resistance *in vivo* results from sources outside of background synaptic activity. To test this hypothesis, we conducted intracellular recordings *in vivo* and used a secondary pipette in close proximity to apply TTX (50 μM) to block local spike activity and eliminate synaptic-mediated input fluctuations.

**Figure 8.**
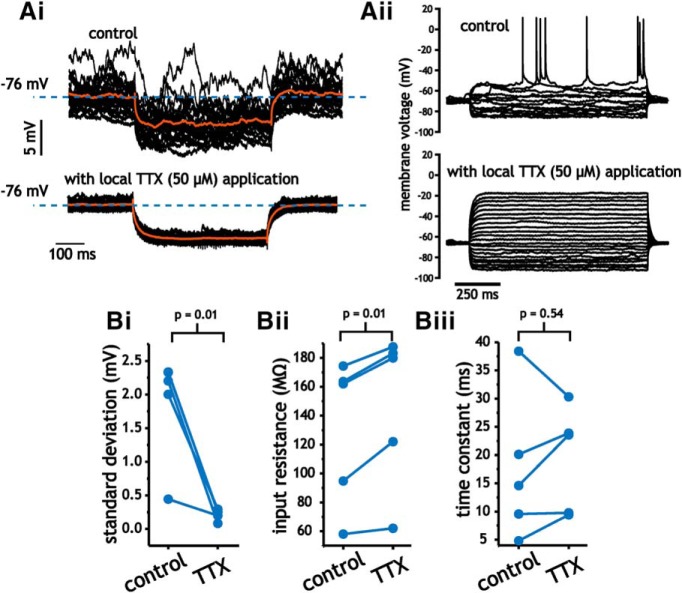
Silencing network activity with TTX minimally impacts average membrane resistance and time constant values measured at rest (−60 to −75 mV). ***A***, Pyramidal cell membrane voltage responses to fixed-size hyperpolarizing current steps in the absence (top panel) or presence (lower panel) of local TTX application. Average response is denoted in red. Note that TTX application was verified by the inability for pyramidal cells to generate spikes at depolarized (>-20 mV) membrane voltages (***Aii***). ***B***, Comparison of membrane voltage standard deviation (i), membrane resistance (ii), and time constants (iii) from paired data generated before and after application of TTX.

We measured input resistance at rest using the average membrane voltage response to 25 repeated presentations of a small (25–50 pA) hyperpolarizing current step (Fig. 8*A*). Membrane resistance was measured at the same membrane voltage before and after TTX application. Within minutes of applying TTX the presence of membrane voltage fluctuations was visibly reduced (Fig. 8*Ai*) and pyramidal cells were no longer capable of generating spikes (Fig. 8*Aii*). Application of TTX significantly decreased the standard deviation of membrane voltage traces (1.86 ± 0.80 vs 0.21 ± 0.08 mV, paired Student’s *t* test, *p* = 0.01, *n* = 5; Fig. 8*Bi*). Membrane input resistance values also changed significantly (131 ± 51.3 vs 147 ± 54.5 MΩ, paired Student’s *t* test, *p* = 0.01, *n* = 5; Fig. 8*Bii*). Although significant, the change was small and indicated an approximate change in average membrane conductance of 0.8 nS. Correspondingly, time constants remained unchanged (19 ± 9.3 vs 18 ± 13 ms, paired Student’s *t* test, *p* = 0.79, *n* = 5; Fig. 8*Biii*). Consistent with our inability to reproduce key features of *in vivo* pyramidal cell in slices using dynamic clamp, these results suggest that the lower input resistance of layer 2/3 mouse somatosensory pyramidal cells *in vivo* is not the product of background synaptic activity generating a steady high conductance state.

## Discussion

Compared to slices, pyramidal cells in mouse somatosensory cortex *in vivo* had reduced membrane input resistance, more depolarized spike voltage threshold, greater spike frequency adaptation, and lower gain. Our results using dynamic clamp indicate that an increase in membrane conductance can fundamentally alter the integration properties of pyramidal cells. Adding conductance, however, changed the majority of measured properties in a manner that was inconsistent with our observations *in vivo*; the *f-V* curve, spike frequency adaptation, and the increase in subthreshold resistance with depolarization were not captured through an increase in membrane conductance. Overall, these results indicate that integration properties of mouse somatosensory pyramidal cells in layer 2/3 *in vivo* fundamentally differ from those in slices, and that these differences are not the result of a high conductance state brought about through background synaptic activity. In our reasoning, these intrinsic differences could arise from neuromodulatory changes and expression controlled through genes.

### Comparisons with previous *in vivo* and slice recordings in S1

Our values of *f-I* curve gain, spike rate of rise, and subthreshold membrane input resistance are similar to those acquired in previous recordings of S1 layer 2/3 pyramidal cells in anesthetized and awake mice (Waters and Helmchen, 2006; Yamashita et al., 2013). Although previous *in vivo* work has indicated narrower spike half-widths in layer 2/3 pyramidal cells (Yamashita et al., 2013), these measures are still significantly broader than our slice data. The statistical and spectral properties of spontaneously generated membrane voltage fluctuations at rest were also similar to those in previous studies under similar conditions (Crochet and Petersen, 2006), which were dominated by low frequency (<10 Hz) power, and positively skewed unimodal distributions.

More detailed comparisons of our pyramidal cell slice and *in vivo* data with those of previous slice recordings are made difficult by two factors. First, a majority of past slice work measuring input-output responses and spike parameters in somatosensory cortex have been done in juvenile (2-4 weeks) mice (Goldberg et al., 2008; Avermann et al., 2012; Economo and White, 2012; Guan et al., 2015). In contrast, we focused on fully mature animals between two and six months of age. Second, different species of rodents have been used in past slice recordings (McCormick et al., 1985; Maravall et al., 2004), which include mice, guinea pigs, and rats.

Pyramidal cells in mouse somatosensory cortex have also been shown to consist of different functional subtypes. For example, within layer 2/3 of barrel cortex, pyramidal cells projecting to different regions are associated with different electrophysiological properties (Yamashita et al., 2013). Although significant, these differences are much smaller than the large differences we observed in membrane resistance, subthreshold *I-V* curves, spike shape, and spike frequency adaptation. Other studies have noted more obvious differences between pyramidal cells, such as the ability to generate a very distinct transient or oscillatory bursting behavior (Jacob et al., 2012). In our recordings, we did not come across any pyramidal cells *in vivo* (*n* = 22) or in slices (*n* = 19) with a phenotype consistent with any form of bursting behavior. This may be, in part, because CaMKIIa is not expressed in all pyramidal cells in layer 2/3 (Wang et al., 2013), and hence exclude those with a bursting phenotype. Alternatively, this could be due to our recordings being limited to a cortical depth between 100 and 200 μm.

### Potential sources of increased membrane conductance under *in vivo* conditions

Low pyramidal cell membrane input resistance and time constant values *in vivo* are consistent with models of cortex in which a high conductance state at the cell body is established through high levels of balanced excitatory and inhibitory synaptic activity (Paré et al., 1998; Destexhe and Paré, 1999; Destexhe et al., 2003; Rudolph et al., 2007).

Application of TTX, however, indicates that the presence of background synaptic activity does not substantially alter average membrane input resistance and time constant values in mouse somatosensory pyramidal cells. Our measures of membrane properties before and after TTX application occurred within a short time frame (<10 min). Changes associated with TTX, therefore, likely represent changes resulting from the absence of faster ionotropic receptor activity (e.g., AMPA and GABA_A_ receptors), rather than slower acting neuromodulatory factors. Nevertheless, the effects of neuromodulatory block by TTX cannot be ruled out.

Differences between our results and past intracellular recordings could result from multiple sources. First, under awake states, we did not observe up-down state transitions as observed by Paré et al. (1998). Up-states likely represent moments of intense and sustained synaptic activity that increase membrane conductance. Second, differences in cortical region and species (cat parietal vs mouse somatosensory) could account for these differences. Superficial layers of cortex may also express lower levels of intense synaptic activity. Intracellular data indicating large contributions to membrane conductance from synaptic inputs have come from recordings conducted in deeper layers of cortex (Paré et al., 1998; Destexhe and Paré, 1999; Reig et al., 2015). Thus, species, layer, and cortical region likely contribute to differences between our findings and past work.

Awake states have also been divided into two broad categories based on whether the animal is actively engaged in sensory processing or not (Crochet and Petersen, 2006; Poulet and Petersen, 2008; Tan et al., 2014). Under quiet conditions, as in our recordings, membrane voltage traces express large synchronous depolarizations, while during sensory processing membrane voltage traces are more desynchronized, depolarized, and express a Gaussian distribution. Depolarized and desynchronized states are likely associated with a higher membrane conductance than quiet states (Tan et al., 2014). Since our recordings were conducted during quiet states, the small change in membrane input resistance associated with silencing synaptic activity could be related to the relative absence of intense synaptic-mediated background conductance. Furthermore, a comparison of membrane input resistance across different studies suggests that the desynchronized state is often associated with lower absolute membrane input resistance than those reported in this study. For example, in cat layer five neurons, input resistance during awake states generating desynchronized LFP activity was reported at ∼20 MΩ (Rudolph et al., 2007), while in rats expressing similar LFP profiles resistance was also ∼20 MΩ (Altwegg-Boussac et al., 2014).

Nevertheless, studies using intracellular patch recordings of layer 2/3 pyramidal cell in S1 of rats during anesthetized states generating up-down state transitions have reported sparse synaptic inputs that contribute a small change in conductances (∼3 nS) during up states (Waters and Helmchen, 2006). Similarly, measures under quiet and whisking states, which are associated with synchronized (low conductance) and desynchronized (high conductance) LFP activity, respectively, have shown small (∼90 vs 75 MΩ, approximate difference of 3 nS) changes in membrane resistance values (Crochet and Petersen, 2006). Although these values are greater than the changes we observed when silencing synaptic-mediated voltage fluctuations, they are significantly smaller than those reported in cat association cortex. For example, Rudolph et al. (2007) reported changes in conductance ranging from 5–170 nS under awake conditions associated with the onset of desynchronized activity in the LFP. Differences between rodents and cats under these conditions could be due to differences in the density of synaptic innervation associated with different cortical regions and species.

Using high levels of pentobarbital, Altwegg-Boussac et al. (2014) were able to silence synaptic activity and compare input resistance with values attained using fentanyl, which generated desynchronized membrane voltage fluctuations similar to a high conductance state under awake conditions. In their case, no significant difference in membrane input resistance between the two conditions was measured despite a significant reduction in membrane voltage fluctuations with pentobarbital (Altwegg-Boussac et al., 2014). Nevertheless, the desynchronized state generated significantly lower membrane input resistance than more synchronized states associated with lower levels of pentobarbital.

Measures comparing quiet and whisking states, which are associated with different forms of synaptic activity, have shown small changes in membrane resistance values (Crochet and Petersen, 2006). Accordingly, it may be that somatosensory mouse neurons *in vivo* express low membrane resistance that is somewhat independent of background AMPAergic and GABAergic synaptic activity; synaptic inputs in this region may constitute a small increase in membrane conductance relative to the intrinsic membrane conductance value.

A distinct possibility is that membrane voltage fluctuations under our conditions arise from excitatory inputs located in dendrites. In fact, a detailed study using artificially-generated synaptic inputs at dendrites using dynamic clamp found that the ability to reconstruct dendritic generated conductances at the soma is highly compromised even in the presence of intracellular solutions used to improve voltage clamp (e.g., QX-314; Williams and Mitchell, 2008)

The significant changes in membrane input resistance with voltage observed *in vivo* also suggests that mouse layer 2/3 pyramidal cells *in vivo* are not exposed to a high conductance state established through synaptic-based linear conductance changes. Although differences in input resistance at *rest* can be reconciled using artificial linear conductance delivered using dynamic clamp, our analyses of *I-V* curves point to a more complex difference between *in vivo* and slices. In our dynamic clamp experiments, a substantial increase in linear conductance, similar to mean conductance changes expected from high levels of AMPAergic and GABAergic background synaptic activity (Destexhe et al., 2003), eliminated the voltage dependence of subthreshold membrane input resistance (Fig. 6*D*). This scenario linearizes the subthreshold *I-V* curve (Fig. 6*D*), a feature inconsistent with our measures of *I-V* curves *in vivo*. Blocking synaptic activity through local TTX also supports this interpretation (Fig. 8). Overall, this suggests that synaptic inputs in mouse layer 2/3 somatosensory cortex under quiet and awake conditions generate sparse synaptic activity that is unable to significantly linearize the subthreshold *I-V* curve.

The extracellular ion environment could also differ from those in slices and impact neuronal membrane and spike output properties. These differences could be accentuated during periods of spike generation and lead to the accumulation of ions in the extracellular space that alter spike output.

Neuromodulators, which can influence voltage-gated conductances (Cantrell and Catterall, 2001), and have been shown to alter neural activity *in vivo* (Constantinople and Bruno, 2011), seem like a more plausible candidate mechanism for the observed differences between slice and *in vivo* pyramidal cells. These factors should be a focus of future studies looking into mechanisms that establish and modulate *in vivo* integration properties.

### Increased membrane conductance and neuronal spike output modulation

As shown here, as well as in previous work, increasing membrane conductance can alter neuronal excitability through mechanisms distinct from the expected changes in membrane time constant. For example, in medial entorhinal cortical stellate cells, increasing conductance significantly reduces membrane resonance and subthreshold oscillations, and can also eliminate oscillatory spike trains at theta frequencies (Fernandez and White, 2008). It has been suggested that this explains the absence of membrane potential oscillations at theta in intracellular recordings of stellate cells in awake mice (Schmidt-Hieber and Häusser, 2013). Similarly, in CA1 pyramidal cells, increasing membrane conductance alters peak spike phase-locking frequency (Broicher et al., 2012), as well as the overall integration behavior of spiking (Prescott et al., 2006).

A consequence of increasing membrane conductance in neurons is a depolarizing shift in spike voltage threshold (Prescott et al., 2006, 2008; Fernandez and White, 2010; Platkiewicz and Brette, 2010). The process of spike generation and crossing threshold involves positive feedback associated with Na^+^ conductance that depolarizes membrane voltage and increases Na^+^ conductance activation, resulting in the rapid depolarization associated with the initial phase of the spike (Brette and Gerstner, 2005; Platkiewicz and Brette, 2010). Increasing membrane conductance reduces the depolarization associated with activation of a set amount of Na^+^ conductance; it takes greater depolarization and Na^+^ current to achieve the sustained positive-feedback for spike initiation. As a result of the depolarizing shift in threshold, other voltage-gated factors, such as Na^+^ current inactivation (Fernandez and White, 2010; Chizhov et al., 2014) and K^+^ current activation (Prescott et al., 2006), can be recruited that alter overall spike output. Regardless of whether this increase results from ongoing background synaptic activity or neuromodulators, it suggests that neurons can fundamentally change their spike integration properties through any processes that increase membrane conductance at the cell body.

### Voltage fluctuations and gain modulation

Some of the differences observed between slice and *in vivo* pyramidal cells could arise from the presence of significant synaptic-mediated voltage fluctuations present *in vivo*, which are largely absent in slices. Artificially-generated random voltage fluctuations in slice neurons can reduce *f-I* curve gain and lower the current and voltage threshold for spiking (Chance et al., 2002; Destexhe et al., 2003; Mitchell and Silver, 2003; Higgs et al., 2006; Silver, 2010). The spectral and statistical properties of intracellular membrane voltage fluctuations we observed under awake conditions, however, differed significantly from those used in models of background synaptic activity. Like previous intracellular recordings in layer 2/3 mouse somatosensory cortical pyramidal cells (Crochet and Petersen, 2006), we measured voltage fluctuations dominated by low frequency power and positive skews, which are consistent with sparse and synchronous volleys of synaptic input (Tan et al., 2014). Because of this difference, the synaptic activity in layer 2/3 mouse somatosensory cortex may not impact input-output responses in the same manner as those established using artificial synaptic inputs in past slice studies. In addition, the depolarized spike voltage thresholds, right-shifted *f-V* curves, and reduced spike firing ranges observed in our recordings of pyramidal cells *in vivo* (Fig. 5*Aii*) are incompatible with modulation by voltage fluctuations, which have been shown to significantly hyperpolarize mean voltage values associated with spiking and increase the firing range (Mitchell and Silver, 2003; Haider and McCormick, 2009; Silver, 2010; Fernandez et al., 2011). In particular, the introduction of membrane voltage fluctuations is expected to left-shift (hyperpolarize) the *f-V* curve. Measures *in vivo*, however, indicated right-shifted *f-V* curves relative to those acquired in slices. Further, introducing noisy current fluctuations has been documented to increase the dynamic range of spike input-output curves (Prescott and De Koninck, 2003; Higgs et al., 2006) and reduce spike frequency adaptation (Fernandez et al., 2011). Dynamic range of pyramidal cells *in vivo*, however, were smaller and spike frequency adaptation greater than what was observed in slices. Differences between the subthreshold *I-V* curves *in vivo* and slices in mouse somatosensory neurons are unlikely to arise from voltage fluctuations. The introduction of noisy fluctuations alone (no conductance component) does not impact the subthreshold *I-V* curve; noisy fluctuations impact input-output responses through spike threshold and *f-I* curve nonlinearities (Chance et al., 2002; Mitchell and Silver, 2003; Prescott and De Koninck, 2003). The addition of noise with a conductance component would linearize the subthreshold *I-V* curve, which is inconsistent with *in vivo* measures. Finally, there is little evidence or plausible way of noise causing the large changes in spike shape. As with our dynamic clamp experiments, these observations suggest that differences between pyramidal cells under the two experimental conditions in mouse somatosensory pyramidal cells stem from factors outside of synaptic-mediated changes in membrane conductance or noise.

## Synthesis

Reviewing Editor: Leonard Maler, University of Ottawa Decisions are customarily a result of the Reviewing Editor and the peer reviewers coming together and discussing their recommendations until a consensus is reached. When revisions are invited, a fact-based synthesis statement explaining their decision and outlining what is needed to prepare a revision will be listed below. The following reviewer(s) agreed to reveal their identity: Alain Destexhe.

Editorial comments

Both reviewers find that the Ms is greatly improved and is now potentially suitable for publication in eNeuro.

The first reviewer has only fairly minor concerns to be addressed.

The second reviewer, however, still needs clarification on brain states and has included figures from published papers to clarify what is still required.

I fully agree with the points raised by both reviewers and, especially, by the cogent arguments of the second reviewer.

Reviewer #1

Overall, I think the authors have done a good job revising the paper. It flows much better now that the material on interneurons has been removed. My original set of concerns have been addressed for the most part. According to my interpretation, the results show that pyramidal neurons in L2/3 of mouse S1 cortex are in a high-conductance state in vivo, but not because of background synaptic input. Instead, other unidentified factors are necessary to account for the pattern of differences in vivo vs. slice. This conclusion is based on the observation that introducing an Ohmic leak conductance, despite partially accounting for some differences, actually exacerbates other differences. And nor did ttx application in vivo have much effect. Given the modest average depolarization, small voltage fluctuations, and lack of spontaneous spiking observed in vivo, it is not so surprising that those neurons are not bombarded by synaptic input, and indeed that is consistent with several past studies but is perhaps forgotten when people equate in vivo conditions with strong background synaptic activity (since the network is intact). This study does not disprove that neurons in other layers/areas are bombarded by strong synaptic input, and nor does it address whether additional differences exist for those neurons when considering in vivo vs slice conditions. It took me a number of readings to converge on this interpretation (which I hope is correct), but the difficulty makes me wonder if there is still room to improve the clarity of the writing with respect to the big picture. 

I have a handful of other concerns: 

-There is no ethics statement, at least I didn't see one in the Materials and Methods section. 

-The comparison with Constantinople and Bruno (2011) is a misleading. Although they refer to their rats as awake, they were all sedated with fentanyl, so the lack of habituation was not really an issue for them. 

-What was the spontaneous firing rate in the cells recorded in vivo? I realize that that number is often quite low (e.g. Fig 3 in O'Connor et al. Neuron 2010) but I think it is helpful to report this in order to compare between studies (including those involving single unit extracellular recordings) and is certainly relevant for comparing between different layers, cortical areas, etc. 

-I still think it is wrong to report the mean and standard deviation (or SEM) to describe data that are not normally distributed. The median and interquartile range could be reported in the text, and would more accurately describe the data, but I defer to the editor and journal policy on how they want that done. 

-Numbering on the y-axis in Fig 3Ai is messed up. 

Reviewer #2

The paper was well improved, and the authors did a great job in clarifying many of the points I raised. 

There still remains a fundamental point that is unclear. The authors seem to record in ‘quiet awake’ conditions, , as they further clarified in their new description. They state that these are not ‘Up and Down’ states, using a definition of Up/Down which would require bimodality. That's fine. 

However, the relevant definition of brain state is not on bimodality, but it must be based on the Vm and LFP/EEG behavior. To clarify this, I attach two figures of previous publications, the first one from rats (Poulet & Petersen, Nature 2008) and the second one from monkey (Tan et al., Nature 2014). In both cases, you see that when the animal is in a non-processing state, the Vm oscillates between -70 and -50 mV. These oscillations are also seen in the LFP and this is what I was previously calling Up/Down states. Besides terminology, it shows that when the animal is processing sensory inputs or is whisking, the oscillations disappear, the LFP switches to desynchronized, and the Vm stays around -55 mV. These are really clear changes of ‘brain state’. 

The problem is that from the LFP provided in the new Fig 1, one cannot see clearly if this is the same dynamics as in previous studies. The Vm dynamics and the Vm-LFP relations, which are clear-cut markers of brain state, are not clearly visible in the figure. Could the authors expand the first 5-10 seconds or so in an inset, to clearly show the Vm and LFP fluctuations, and how Vm and LFPs are related ? 

Assuming that all the experimental conditions here correspond to this quiet awake state with oscillatory Vm, it is not totally clear if the comparison with previous studies can be made so easily. It seems to me that many previous measurements, such as Rudolph et al 2007, were done in a strictly desynchronized EEG state, which does not represent the same brain state as the present measurements. This can also be seen in the attached figure. The measurements of Altwegg-Boussac et al., 2014 seem to show that only this desynchronized-EEG state corresponds to a high-conductance state in the membrane. So this suggests that the authors actually made their measurements in a low-conductance state, which is only one particular state in the awake animal. In this context, these measurements are maybe not contradictory at all with previous studies who measured in a truly high-conductance state with desynchronized EEG. To clarify this, the authors should publish their absolute Rin values (before manipulations), perhaps as a table, and compare these values to previous Rin measurements in awake animals (monkey, cat, rat, mice) and clarify in which EEG state they correspond to (oscillatory or desynchronized). This information would considerably help the reader to make sense of the measurements and how to relate them to previous measurements. 

## Author Response

Editorial comments

Both reviewers find that the Ms is greatly improved and is now potentially suitable for publication in eNeuro.

The first reviewer has only fairly minor concerns to be addressed.

The second reviewer, however, still needs clarification on brain states and has included figures from published papers to clarify what is still required.

I fully agree with the points raised by both reviewers and, especially, by the cogent arguments of the second reviewer.

Reviewer #1

Overall, I think the authors have done a good job revising the paper. It flows much better now that the material on interneurons has been removed. My original set of concerns have been addressed for the most part. According to my interpretation, the results show that pyramidal neurons in L2/3 of mouse S1 cortex are in a high-conductance state in vivo, but not because of background synaptic input. Instead, other unidentified factors are necessary to account for the pattern of differences in vivo vs. slice. This conclusion is based on the observation that introducing an Ohmic leak conductance, despite partially accounting for some differences, actually exacerbates other differences. And nor did ttx application in vivo have much effect. Given the modest average depolarization, small voltage fluctuations, and lack of spontaneous spiking observed in vivo, it is not so surprising that those neurons are not bombarded by synaptic input, and indeed that is consistent with several past studies but is perhaps forgotten when people equate in vivo conditions with strong background synaptic activity (since the network is intact). This study does not disprove that neurons in other layers/areas are bombarded by strong synaptic input, and nor does it address whether additional differences exist for those neurons when considering in vivo vs slice conditions. It took me a number of readings to converge on this interpretation (which I hope is correct), but the difficulty makes me wonder if there is still room to improve the clarity of the writing with respect to the big picture.

I have a handful of other concerns:

-There is no ethics statement, at least I didn't see one in the Materials and Methods section.

Response:

We have now added an ethics statement. It was initially removed in order to satisfy the double-blind review process.

-The comparison with Constantinople and Bruno (2011) is a misleading. Although they refer to their rats as awake, they were all sedated with fentanyl, so the lack of habituation was not really an issue for them.

Response:

We have removed this statement.

-What was the spontaneous firing rate in the cells recorded in vivo? I realize that that number is often quite low (e.g. Fig 3 in O'Connor et al. Neuron 2010) but I think it is helpful to report this in order to compare between studies (including those involving single unit extracellular recordings) and is certainly relevant for comparing between different layers, cortical areas, etc.

Response:

Using whole-cell recording we found that most cells did not fire. This is likely due to a hyperpolarizing effect of our intracellular solution. However, using imaging of GCaMP6f (virally expressed) and deconvolution analysis to extract firing rates from individual cells, we found average firing rates of ~ 2 Hz, which are similar to those reported by O'Connor et al. (2010)(~3 Hz using imaging), but higher than those of Crochet and Petersen (2006) (~1 Hz using whole cell recordings). Overall, these rates are within the values reported previously in Layer 2/3 We now report this in the Result section and have added a new panel to Figure 2 (panel D) that shows these data.

-I still think it is wrong to report the mean and standard deviation (or SEM) to describe data that are not normally distributed. The median and interquartile range could be reported in the text, and would more accurately describe the data, but I defer to the editor and journal policy on how they want that done.

Response:

We now indicate the interquartile range for non-normally distributed data.

-Numbering on the y-axis in Fig 3Ai is messed up.

Reponse:

We have fixed this.

Reviewer #2

The paper was well improved, and the authors did a great job in clarifying many of the points I raised.

There still remains a fundamental point that is unclear. The authors seem to record in ‘quiet awake’ conditions, , as they further clarified in their new description. They state that these are not ‘Up and Down’ states, using a definition of Up/Down which would require bimodality. That's fine.

However, the relevant definition of brain state is not on bimodality, but it must be based on the Vm and LFP/EEG behavior. To clarify this, I attach two figures of previous publications, the first one from rats (Poulet & Petersen, Nature 2008) and the second one from monkey (Tan et al., Nature 2014). In both cases, you see that when the animal is in a non-processing state, the Vm oscillates between -70 and -50 mV. These oscillations are also seen in the LFP and this is what I was previously calling Up/Down states. Besides terminology, it shows that when the animal is processing sensory inputs or is whisking, the oscillations disappear, the LFP switches to desynchronized, and the Vm stays around -55 mV. These are really clear changes of ‘brain state’.

The problem is that from the LFP provided in the new Fig 1, one cannot see clearly if this is the same dynamics as in previous studies. The Vm dynamics and the Vm-LFP relations, which are clear-cut markers of brain state, are not clearly visible in the figure. Could the authors expand the first 5-10 seconds or so in an inset, to clearly show the Vm and LFP fluctuations, and how Vm and LFPs are related ?

Response:

We have now expanded the traces shown in Figure 1 so that the last 15 seconds are shown. As suggested by the reviewer, synchronous volleys of activity are clearly discernable. Also, the LFP shows a peak in the power spectra which is consistent with a synchronous and low conductance state described by Tan et al. (2014) and Poulet and Petersen (2008). Finally, the cross-correlation analysis indicates that the LFP and Vm are significantly correlated, which is both consistent with previous recordings under quiet and awake conditions, as well with synchronous activity in cortex. In the results section we now refer to the activity we observed as synchronous and low conductance, and cite the papers as indicating a synchronous state consistent with previous studies recording under quiet and awake conditions. We thank the reviewer for this improvement.

Assuming that all the experimental conditions here correspond to this quiet awake state with oscillatory Vm, it is not totally clear if the comparison with previous studies can be made so easily. It seems to me that many previous measurements, such as Rudolph et al 2007, were done in a strictly desynchronized EEG state, which does not represent the same brain state as the present measurements. This can also be seen in the attached figure. The measurements of Altwegg-Boussac et al., 2014 seem to show that only this desynchronized-EEG state corresponds to a high-conductance state in the membrane. So this suggests that the authors actually made their measurements in a low-conductance state, which is only one particular state in the awake animal.

Response:

We agree that the desynchronized state is likely a higher conductance state. We now state in the Results section that our recordings are consistent with a low conductance and synchronous state as described by Tan et al. (2014). We also describe in the Discussion section that our results are different from those of Rudolph et al. 2007 because our measures were taken in a lower conductance state associated with synchronous LFP activity.

A direct comparison with those of Altwegg-Boussac et al 2014 is difficult as anesthesia, irrespective of whether the LFP/EEG indicates synchronous or desynchronized activity, has the potential to alter membrane resistance through neuromodulation. Nevertheless, we agree that the depolarized and desynchronized state associated with a high conductance reported by Altwegg-Boussac et al 2014 is consistent with those reported by Tan et al (2014) and Poulet and Petersen (2008). We now cite the paper in this context, and report in the Discussion that our measures are likely from a lower conductance state.

In this context, these measurements are maybe not contradictory at all with previous studies who measured in a truly high-conductance state with desynchronized EEG. To clarify this, the authors should publish their absolute Rin values (before manipulations), perhaps as a table, and compare these values to previous Rin measurements in awake animals (monkey, cat, rat, mice) and clarify in which EEG state they correspond to (oscillatory or desynchronized). This information would considerably help the reader to make sense of the measurements and how to relate them to previous measurements.

Response:

In the revised discussion, we emphasize that our data were not recorded in the high-conductance state. We also compare values of absolute Rin between cat, rats and mice neurons to make the point.

Nevertheless, a comparison of absolute membrane resistance is limited since all these studies have been carried in different regions (association cortex, S1, V1), species (cats, rats, mice), and used different recording methods (patch vs. sharp). As discussed in our previous response, there are numerous confounds to interpreting a comparison in the input resistance between values reported in different species and regions as the biophysical properties of neurons are known to differ between regions within the same species, and are affected by the recording method. These differences likely arise from differences in the density of synaptic innervation between different cortical regions and species. We now state this in the Discussion.

For these reasons, we have also added a section to the Discussion that compares the change in membrane input resistance with TTX with those of others that have measured changes in resistance resulting from sensory input or changes in brain state. Thus, in addition to comparing absolute input resistance, we provide measures of net changes in conductance associated with the onset of intense synaptic activity/desynchronized states. Together, these points suggest that differences between our results and past studies are likely related to brain state associated with synchronized/desynchronized LFPs, as well as animal/cortical region.

## References

[B1] Altwegg-Boussac T, Chavez M, Mahon S, Charpier S (2014) Excitability and responsiveness of rat barrel cortex neurons in the presence and absence of spontaneous synaptic activity in vivo. J Physiol 592:3577–3595. 10.1113/jphysiol.2013.270561 24732430 PMC4229349

[B2] Anderson JS, Lampl I, Gillespie DC, Ferster D (2000) The contribution of noise to contrast invariance of orientation tuning in cat visual cortex. Science 290:1968–1972. 11110664 10.1126/science.290.5498.1968

[B3] Avermann M, Tomm C, Mateo C, Gerstner W, Petersen CCH (2012) Microcircuits of excitatory and inhibitory neurons in layer 2/3 of mouse barrel cortex. J Neurophysiol 107:3116–3134. 10.1152/jn.00917.2011 22402650

[B4] Bernander O, Douglas RJ, Martin KA, Koch C (1991) Synaptic background activity influences spatiotemporal integration in single pyramidal cells. Proc Natl Acad Sci USA 88:11569–11573. 1763072 10.1073/pnas.88.24.11569 PMC53177

[B5] Bettencourt JC, Lillis KP, Stupin LR, White JA (2008) Effects of imperfect dynamic clamp: computational and experimental results. J Neurosci Methods 169:282–289. 10.1016/j.jneumeth.2007.10.009 18076999 PMC2387131

[B6] Brette R, Gerstner W (2005) Adaptive exponential integrate-and-fire model as an effective description of neuronal activity. J Neurophysiol 94:3637–3642. 10.1152/jn.00686.2005 16014787

[B7] Broicher T, Malerba P, Dorval AD, Borisyuk A, Fernandez FR, White JA (2012) Spike phase locking in CA1 pyramidal neurons depends on background conductance and firing rate. J Neurosci 32:14374–14388. 10.1523/JNEUROSCI.0842-12.2012 23055508 PMC3506380

[B8] Cantrell AR, Catterall WA (2001) Neuromodulation of Na+ channels: an unexpected form of cellular plasticity. Nat Rev Neurosci 2:397–407. 10.1038/35077553 11389473

[B9] Chance FS, Abbott LF, Reyes AD (2002) Gain modulation from background synaptic input. Neuron 35:773–782. 12194875 10.1016/s0896-6273(02)00820-6

[B10] Chizhov AV, Smirnova EY, Kim KK, Zaitsev AV (2014) A simple Markov model of sodium channels with a dynamic threshold. J Comput Neurosci 37:181–191. 10.1007/s10827-014-0496-6 24469252

[B11] Constantinople CM, Bruno RM (2011) Effects and mechanisms of wakefulness on local cortical networks. Neuron 69:1061–1068. 10.1016/j.neuron.2011.02.040 21435553 PMC3069934

[B12] Crochet S, Petersen CC (2006) Correlating whisker behavior with membrane potential in barrel cortex of awake mice. Nat Neurosci 9:608–610. 10.1038/nn1690 16617340

[B13] Crochet S, Poulet JFA, Kremer Y, Petersen CCH (2011) Synaptic mechanisms underlying sparse coding of active touch. Neuron 69:1160–1175. 10.1016/j.neuron.2011.02.022 21435560

[B14] Cudmore RH, Turrigiano GG (2004) Long-term potentiation of intrinsic excitability in LV visual cortical neurons. J Neurophysiol 92:341–348. 10.1152/jn.01059.2003 14973317

[B15] Dembrow N, Johnston D (2014) Subcircuit-specific neuromodulation in the prefrontal cortex. Front Neural Circuits 8:54. 24926234 10.3389/fncir.2014.00054 PMC4046580

[B16] Desai NS, Rutherford LC, Turrigiano GG (1999) Plasticity in the intrinsic excitability of cortical pyramidal neurons. Nat Neurosci 2:515–520. 10.1038/9165 10448215

[B17] Destexhe A, Paré D (1999) Impact of network activity on the integrative properties of neocortical pyramidal neurons in vivo. J Neurophysiol 81:1531–1547. 10.1152/jn.1999.81.4.1531 10200189

[B18] Destexhe A, Rudolph M, Fellous JM, Sejnowski TJ (2001) Fluctuating synaptic conductances recreate in vivo-like activity in neocortical neurons. Neuroscience 107:13–24. 11744242 10.1016/s0306-4522(01)00344-x PMC3320220

[B19] Destexhe A, Rudolph M, Paré D (2003) The high-conductance state of neocortical neurons in vivo. Nat Rev Neurosci 4:739–751. 10.1038/nrn1198 12951566

[B20] Economo MN, White JA (2012) Membrane properties and the balance between excitation and inhibition control gamma-frequency oscillations arising from feedback inhibition. PLoS Comput Biol 8:e1002354. 10.1371/journal.pcbi.1002354 22275859 PMC3261914

[B21] Fernandez FR, White JA (2008) Artificial synaptic conductances reduce subthreshold oscillations and periodic firing in stellate cells of the entorhinal cortex. J Neurosci 28:3790–3803. 10.1523/JNEUROSCI.5658-07.2008 18385337 PMC6671103

[B22] Fernandez FR, White JA (2010) Gain control in CA1 pyramidal cells using changes in somatic conductance. J Neurosci 30:230–241. 10.1523/JNEUROSCI.3995-09.2010 20053905 PMC2865889

[B23] Fernandez FR, Broicher T, Truong A, White JA (2011) Membrane voltage fluctuations reduce spike frequency adaptation and preserve output gain in CA1 pyramidal neurons in a high-conductance state. J Neurosci 31:3880–3893. 10.1523/JNEUROSCI.5076-10.2011 21389243 PMC3483084

[B24] Ferrante M, Shay CF, Tsuno Y, William Chapman G, Hasselmo ME (2017) Post-inhibitory rebound spikes in rat medial entorhinal layer II/III principal cells: in vivo, in vitro, and computational modeling characterization. Cereb Cortex 27:2111–2125. 10.1093/cercor/bhw058 26965902 PMC5963826

[B25] Goldberg EM, Clark BD, Zagha E, Nahmani M, Erisir A, Rudy B (2008) K+ channels at the axon initial segment dampen near-threshold excitability of neocortical fast-spiking GABAergic interneurons. Neuron 58:387–400. 10.1016/j.neuron.2008.03.003 18466749 PMC2730466

[B26] Guan D, Armstrong WE, Foehring RC (2015) Electrophysiological properties of genetically identified subtypes of layer 5 neocortical pyramidal neurons: Ca2+ dependence and differential modulation by norepinephrine. J Neurophysiol 113:2014–2032. 10.1152/jn.00524.2014 25568159 PMC4416592

[B27] Haider B, McCormick DA (2009) Rapid neocortical dynamics: cellular and network mechanisms. Neuron 62:171–189. 10.1016/j.neuron.2009.04.008 19409263 PMC3132648

[B28] Hartigan JA, Hartigan PM (1985) The dip test of unimodality. Ann Stat 13:70–84. 10.1214/aos/1176346577

[B29] Higgs MH, Slee SJ, Spain WJ (2006) Diversity of gain modulation by noise in neocortical neurons: regulation by the slow afterhyperpolarization conductance. J Neurosci 26:8787–8799. 10.1523/JNEUROSCI.1792-06.2006 16928867 PMC6674385

[B30] Hô N, Destexhe A (2000) Synaptic background activity enhances the responsiveness of neocortical pyramidal neurons. J Neurophysiol 84:1488–1496. 10.1152/jn.2000.84.3.1488 10980021

[B31] Hodgkin AL, Katz B (1949) The effect of sodium ions on the electrical activity of the giant axon of the squid. J Physiol 108:37–77. 18128147 10.1113/jphysiol.1949.sp004310 PMC1392331

[B32] Holt GR, Softky WR, Koch C, Douglas RJ (1996) Comparison of discharge variability in vitro and in vivo in cat visual cortex neurons. J Neurophysiol 75:1806–1814. 10.1152/jn.1996.75.5.1806 8734581

[B33] Hondeghem LM (1978) Validity of Vmax as a measure of the sodium current in cardiac and nervous tissues. Biophys J 23:147–152. 10.1016/S0006-3495(78)85439-3 667303 PMC1473556

[B34] Jacob V, Petreanu L, Wright N, Svoboda K, Fox K (2012) Regular spiking and intrinsic bursting pyramidal cells show orthogonal forms of experience-dependent plasticity in layer V of barrel cortex. Neuron 73:391–404. 10.1016/j.neuron.2011.11.034 22284191 PMC3524456

[B35] Kumar A, Schrader S, Aertsen A, Rotter S (2008) The high-conductance state of cortical networks. Neural Comput 20:1–43. 10.1162/neco.2008.20.1.1 18044999

[B36] Maravall M, Stern EA, Svoboda K (2004) Development of intrinsic properties and excitability of layer 2/3 pyramidal neurons during a critical period for sensory maps in rat barrel cortex. J Neurophysiol 92:144–156. 10.1152/jn.00598.2003 14973314

[B37] McCormick DA, Connors BW, Lighthall JW, Prince DA (1985) Comparative electrophysiology of pyramidal and sparsely spiny stellate neurons of the neocortex. J Neurophysiol 54:782–806. 10.1152/jn.1985.54.4.782 2999347

[B38] Mitchell SJ, Silver RA (2003) Shunting inhibition modulates neuronal gain during synaptic excitation. Neuron 38:433–445. 12741990 10.1016/s0896-6273(03)00200-9

[B39] Nadim F, Bucher D (2014) Neuromodulation of neurons and synapses. Curr Opin Neurobiol 29:48–56. 10.1016/j.conb.2014.05.003 24907657 PMC4252488

[B40] O’Connor DH, Peron SP, Huber D, Svoboda K (2010) Neural activity in barrel cortex underlying vibrissa-based object localization in mice. Neuron 67:1048–1061. 20869600 10.1016/j.neuron.2010.08.026

[B41] Okun M, Naim A, Lampl I (2010) The subthreshold relation between cortical local field potential and neuronal firing unveiled by intracellular recordings in awake rats. J Neurosci 30:4440–4448. 10.1523/JNEUROSCI.5062-09.2010 20335480 PMC6634481

[B42] Paré D, Shink E, Gaudreau H, Destexhe A, Lang EJ (1998) Impact of spontaneous synaptic activity on the resting properties of cat neocortical pyramidal neurons in vivo. J Neurophysiol 79:1450–1460. 10.1152/jn.1998.79.3.1450 9497424

[B43] Platkiewicz J, Brette R (2010) A threshold equation for action potential initiation. PLoS Comput Biol 6:e1000850. 10.1371/journal.pcbi.1000850 20628619 PMC2900290

[B44] Poulet JFA, Petersen CCH (2008) Internal brain state regulates membrane potential synchrony in barrel cortex of behaving mice. Nature 454:881–885. 10.1038/nature07150 18633351

[B45] Prescott SA, De Koninck Y (2003) Gain control of firing rate by shunting inhibition: roles of synaptic noise and dendritic saturation. Proc Natl Acad Sci USA 100:2076–2081. 10.1073/pnas.0337591100 12569169 PMC149961

[B46] Prescott SA, Ratté S, De Koninck Y, Sejnowski TJ (2006) Nonlinear interaction between shunting and adaptation controls a switch between integration and coincidence detection in pyramidal neurons. J Neurosci 26:9084–9097. 10.1523/JNEUROSCI.1388-06.2006 16957065 PMC2913017

[B47] Prescott SA, Ratté S, De Koninck Y, Sejnowski TJ (2008) Pyramidal neurons switch from integrators in vitro to resonators under in vivo-like conditions. J Neurophysiol 100:3030–3042. 10.1152/jn.90634.2008 18829848 PMC2604842

[B48] Priebe NJ, Ferster D (2008) Inhibition, spike threshold, and stimulus selectivity in primary visual cortex. Neuron 57:482–497. 10.1016/j.neuron.2008.02.005 18304479

[B49] Reig R, Zerlaut Y, Vergara R, Destexhe A, Sanchez-Vives MV (2015) Gain modulation of synaptic inputs by network state in auditory cortex in vivo. J Neurosci 35:2689–2702. 10.1523/JNEUROSCI.2004-14.2015 25673859 PMC6605611

[B50] Rudolph M, Pospischil M, Timofeev I, Destexhe A (2007) Inhibition determines membrane potential dynamics and controls action potential generation in awake and sleeping cat cortex. J Neurosci 27:5280–5290. 10.1523/JNEUROSCI.4652-06.2007 17507551 PMC6672346

[B51] Schmidt-Hieber C, Häusser M (2013) Cellular mechanisms of spatial navigation in the medial entorhinal cortex. Nat Neurosci 16:325–331. 10.1038/nn.3340 23396102

[B52] Silver RA (2010) Neuronal arithmetic. Nat Rev Neurosci 11:474–489. 10.1038/nrn2864 20531421 PMC4750293

[B53] Softky WR, Koch C (1993) The highly irregular firing of cortical cells is inconsistent with temporal integration of random EPSPs. J Neurosci 13:334–350. 8423479 10.1523/JNEUROSCI.13-01-00334.1993 PMC6576320

[B54] Strichartz G, Cohen I (1978) Vmax as a measure of GNa in nerve and cardiac membranes. Biophys J 23:153–156. 10.1016/S0006-3495(78)85440-X 667304 PMC1473546

[B55] Tan AYY, Chen Y, Scholl B, Seidemann E, Priebe NJ (2014) Sensory stimulation shifts visual cortex from synchronous to asynchronous states. Nature 509:226–229. 10.1038/nature13159 24695217 PMC4067243

[B56] Tsien JZ, Chen DF, Gerber D, Tom C, Mercer EH, Anderson DJ, Mayford M, Kandel ER, Tonegawa S (1996) Subregion- and cell type-restricted gene knockout in mouse brain. Cell 87:1317–1326. 8980237 10.1016/s0092-8674(00)81826-7

[B57] Tsuno Y, Chapman GW, Hasselmo ME (2015) Rebound spiking properties of mouse medial entorhinal cortex neurons in vivo. Eur J Neurosci 42:2974–2984. 10.1111/ejn.13097 26454151 PMC4780755

[B58] Wang X, Zhang C, Szábo G, Sun Q-Q (2013) Distribution of CaMKIIα expression in the brain in vivo, studied by CaMKIIα-GFP mice. Brain Res 1518:9–25. 10.1016/j.brainres.2013.04.042 23632380 PMC3747672

[B59] Waters J, Helmchen F (2006) Background synaptic activity is sparse in neocortex. J Neurosci 26:8267–8277. 10.1523/JNEUROSCI.2152-06.2006 16899721 PMC6673816

[B60] Wester JC, McBain CJ (2014) Behavioral state-dependent modulation of distinct interneuron subtypes and consequences for circuit function. Curr Opin Neurobiol 29:118–125. 10.1016/j.conb.2014.07.007 25058112 PMC4268408

[B61] Williams SR, Mitchell SJ (2008) Direct measurement of somatic voltage clamp errors in central neurons. Nat Neurosci 11:790–798. 10.1038/nn.2137 18552844

[B62] Wolfart J, Debay D, Le Masson G, Destexhe A, Bal T (2005) Synaptic background activity controls spike transfer from thalamus to cortex. Nat Neurosci 8:1760–1767. 10.1038/nn1591 16261132

[B63] Yamashita T, Pala A, Pedrido L, Kremer Y, Welker E, Petersen CCH (2013) Membrane potential dynamics of neocortical projection neurons driving target-specific signals. Neuron 80:1477–1490. 10.1016/j.neuron.2013.10.059 24360548

[B64] Zariwala HA, Madisen L, Ahrens KF, Bernard A, Lein ES, Jones AR, Zeng H (2011) Visual tuning properties of genetically identified layer 2/3 neuronal types in the primary visual cortex of cre-transgenic mice. Front Syst Neurosci 4:162. 10.3389/fnsys.2010.00162 21283555 PMC3028542

